# A reflection on synthesis by extrusion ten years on: achievements, challenges and opportunities for solvent-free, sustainable, continuous chemical manufacturing

**DOI:** 10.1039/d6sc90085b

**Published:** 2026-05-01

**Authors:** Deborah E. Crawford, Stuart L. James

**Affiliations:** a School of Chemistry, University of Birmingham Edgbaston Birmingham B15 2TT UK d.e.crawford@bham.ac.uk; b Birmingham Centre for Mechanochemistry and Mechanical Processing, University of Birmingham Edgbaston Birmingham B15 2TT UK; c School of Chemistry and Chemical Engineering, Queen’s University Belfast David Kier Building, Stranmillis Road Belfast BT9 5AG UK s.james@qub.ac.uk

## Abstract

In 2015, we and colleagues published an article reporting that it was possible to synthesise metal organic frameworks (MOFs) and discrete coordination complexes by Twin Screw Extrusion (TSE) – a remarkably efficient, continuous and potentially scalable synthesis technique that required little or no solvent (D. Crawford, J. Casaban, R. Haydon, N. Giri, T. McNallyand S. L. James, *Chem. Sci.* 2015, **6**, 1645, https://doi.org/10.1039/C4SC03217A). In the years since its publication, this work has had impact across both academia and industry. Here, we reflect on the origins of that study and its place in the rapidly advancing field of mechanochemical synthesis. We also discuss the challenges and opportunities that lie ahead for the further development and implementation of TSE as an enabling and sustainable method for chemical synthesis and manufacturing.

## Abstract 2

In celebration of our 15th anniversary and some of our most popular articles, Deborah Crawford and Stuart James reflect on the area of synthesis by extrusion, building on the first paper published in *Chemical Science* on this topic (James *et al.*, *Chem. Sci.*, 2015, https://doi.org/10.1039/C4SC03217A).

## Graphical abstract

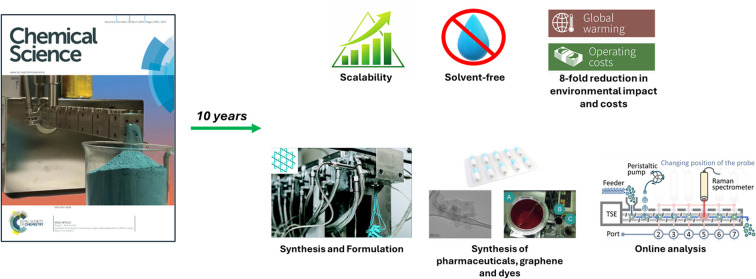

## The context – mechanochemical synthesis by ball milling

In the years leading up to the 2015 publication of ‘Synthesis by extrusion: continuous, large-scale preparation of MOFs using little or no solvent’ (https://doi.org/10.1039/C4SC03217A),^1^ research into mechanochemical synthesis was steadily gaining momentum. Thanks to a growing group of enthusiastic researchers, it was becoming increasingly accepted that “simply” grinding solids together, with a mortar and pestle or by ball milling, with little or no solvent is a viable way to carry out chemical synthesis. This approach was also recognised as being less wasteful and potentially highly economical, using less material and generating less waste than conventional solvent-based methods. Consequently, there was a surge of interest as researchers sought to develop a fundamental understanding of mechanochemical synthesis, including which reactions were, or were not, amenable to this approach, as well as the underlying kinetics and mechanisms. However, a further critical challenge was how to scale up mechanochemical processes. Addressing this issue would be pivotal to the adoption of mechanochemistry in chemical manufacturing and to translating its laboratory-scale advantages into meaningful real-world benefits.

Research into metal organic frameworks (MOFs) was already booming in the early 2000s, with some fascinating results showing that different solvents could result in different framework topologies. This led us to consider what would be the outcome if MOFs were synthesised under solvent-free conditions. Having been inspired at a lecture by Gerd Kaupp at a Royal Society of Chemistry Discussion meeting in 2003, we purchased a Retsch MM200 ball mill, and our investigation into mechanochemical MOF synthesis began.

On an otherwise quiet Friday afternoon, our first speculative experiments were attempted. Remarkably, it was found quite quickly that ball milling a solid metal salt (Cu(OAc)_2_·H_2_O) with a solid organic linker (isonicotinic acid, NC_5_H_4_-4-CO_2_H, INAH), without any added solvent, gave a 3-dimensional porous MOF material [Cu(INA)_2_] quantitatively as a crystalline powder in just 10 minutes.^2^ This result was particularly striking at a time when MOF syntheses typically required hours or even days under solvothermal conditions. Furthermore, this success was extended to Cu_3_(BTC)_2_ (HKUST-1), a MOF that remains of considerable interest due to its excellent gas-adsorption properties. This material was obtained by grinding Cu(OAc)_2_·H_2_O with an alternative linker, H_3_BTC (BTC = 1,3,5-benzenetricarboxylic acid). In both MOFs, the same network topologies were obtained by ball milling as under solution conditions. Most likely, the by-products, AcOH/H_2_O, were important in acting as internally-generated solvent – enabling diffusion of reactants (*via in situ* generated liquid assisted grinding (LAG)) and acting as a pore template. Indeed, additional experiments revealed that leaving a partially reacted mixture in the AcOH vapour by-product enabled the reaction to continue without further milling. In the absence of the vapour, the reaction stopped.

These findings were published in *CrystEngComm* in 2006.^2^ Although this seems to be the first solvent-free mechanochemical synthesis of a porous MOF directly from a metal salt and an organic linker, Braga and Steed had previously independently reported the synthesis of non-porous 1-D coordination polymers by manual grinding in a mortar and pestle,^3*a*^ and related previous work by Bourne *et al.* involved a 1-D coordination polymer ZnBr_2_(pyrazine) being reacted with additional pyrazine in a WIG-L-BUG (a type of small ball mill) to give a 2-D network ZnBr_2_(pyrazine)_2_.^3*b*^ Interestingly, in some very early work on MOF-type materials, Musgrave and Mattson (1968) had prepared metal nitrate-4,4′-bipyridine square networks in a method which involved grinding in a mortar and pestle, although this was done with a large excess of methanol solvent (*i.e.* as a slurry) as well as the 4,4′-bipyridine ligand (50–100 fold excess) and may have occurred substantially in solution.^3*c*^

We extended our initial positive findings by studying an array of some 60 interrelated reactions.^4^ This revealed that this type of reactivity was actually quite general; more often than not, divalent salts of labile first-row *d*-block metals (Ni, Cu, Zn) reacted with various solid bridging N- or O-donor organic ligands by ball milling to give crystalline products within a few minutes. The fact that not all attempts were successful helped to reveal reactivity patterns. Factors favouring reaction included the formation of adventitious internal solvent such as water or acetic acid as by-products, ligands having low melting points and metal salts with more basic anions if the reaction was with an acidic ligand *e.g.* acetates were more reactive than nitrates. These reactivity trends were intuitively reasonable, suggesting that, although unusual, this method of synthesis was amenable to the types of rationale applied in solution phase synthesis. Some previously unknown materials were also obtained as part of this work although their precise crystal structures could not be identified at that stage.

Over the next few years further examples of MOF synthesis by ball milling or manual grinding were reported by a growing number of groups including those of Emmerling,^5^ Friščić,^6^ Mehring^7^ and ourselves.^8^ This extended to the use of insoluble, and normally rather inert, starting materials, such as ZnO.^6*d*^ Intriguingly, as well as being formed by grinding, many MOFs were also found to be quite reactive under ball milling conditions, undergoing structural rearrangements or functionalisation with additional ligands,^9^ for example, drawing strong parallels with the behaviour of organic cocrystals.^10^ The development of *in situ* techniques for monitoring these reactions directly without interrupting the milling process revealed key structural information on the occurrence of intermediate crystalline phases,^6*c*^ for example, as well as the effects of small amounts of added solvent compared to neat grinding. The use of PXRD techniques to determine directly the structures of the obtained materials and reveal subtle structural aspects was also demonstrated.^8*c*^ Overall, this body of work demonstrated mechanochemistry as a versatile approach for accessing both conventional and previously unreported MOF structures, and the intersection between mechanochemistry and MOFs is an active and expanding field of research, as recently reviewed.^11^

Much of the foundations of mechanochemistry in the modern era had previously been laid by pioneers from the 1960s onward, often in the context of extended inorganic materials but also molecular crystals.^12^ The International Mechanochemical Association had been established in 1988, providing an important organisational framework for the field. In parallel, the International Conference on Mechanochemistry and Mechanical Alloying (INCOME) and the International Conference on the Fundamental Bases of Mechanochemical Technologies (Novosibirsk) provided important international platforms for researchers to exchange ideas and foster collaboration. From the late 1990s onward, the field entered a period of rapid development as new possibilities in mechanochemical synthesis emerged and the mechanochemistry community began to expand dramatically. An aspect of this was a shift to include applications in molecular organic synthesis,^13^ MOFs and the synthesis of co-crystals.^14^ Two symposia held in Belfast in 2009 and 2011 brought many of this nascent generation of new researchers together and ultimately resulted in an influential joint review article, which helped to define the field and articulate the key challenges for mechanochemical synthesis at that point in time.^15^ This was soon followed by a larger collection of specialised reviews published in *Chemical Society Reviews*^16^ and an increasing number of international meetings and symposia as the field continued to gain momentum. In turn, the growing field of mechanochemical synthesis positioned itself within the wider discipline of mechanochemistry, encompassing areas such as polymer mechanochemistry,^17^ as well as within the broader framework of green chemistry.

In Belfast, our focus shifted from discovering new MOF materials^18^ to examining the broader scope, advantages, limitations, and underlying mechanisms of mechanochemistry itself, not only in the context of MOFs but also in covalent organic synthesis^19^ and the preparation of solid-supported catalysts.^20^ We consolidated around two overall objectives: (i) to develop a deeper fundamental understanding of mechanochemical reactions, particularly through the formulation of physical reaction models and insight into molecular-scale processes, and (ii) to demonstrate that mechanochemical synthesis could be scaled up to industrially relevant levels.

In addressing our first goal, kinetic monitoring carried out within our group, together with important contributions from Boldyreva and co-workers, led to the development of physical reaction models. In some cases, the reactions proved surprisingly simple or “pseudo-fluid” in nature.^21^ In others, they were more complex, proceeding through distinct rheological transitions in which crystalline molecular powders transformed into rubber-like phases. These transformations had a pronounced impact on reaction kinetics and were described by a “cohesive-state” feedback model.^22^ Molecular dynamics modelling revealed smaller-scale details of the processes occurring during collisions and indentations between organic crystals, including amorphisation and “plasticine-like” behaviour at the molecular level, during which molecular mixing occurred.^23^ This naturally raises the question of whether such molecular-scale plasticine-like behaviour is related to the cohesive phases observed macroscopically during ball-milling reactions. Elucidating relationships between these different size scales remains an open and important challenge in the field (see discussion of rheokinetics below).^24^

Our second goal focused on exploring practical strategies for conducting mechanochemical synthesis at scale, most notably through the use of twin-screw extrusion (TSE), which led to our 2015 publication describing the synthesis of MOFs and coordination complexes by TSE, forming the focus of this mini-review.

## The move to synthesis by extrusion to demonstrate scalability and industrial applicability

Most of the work summarised above was conducted on sub-gram scales using ball mills, occasionally reaching a few grams,^25*a*^ and at the time offered very limited control over temperature. Although the literature includes some examples of successful large-scale milling, there has been scepticism about relying on planetary and tumbler mills as universally applicable methods for large-scale mechanochemistry. Indeed, in our own experience, scaling from 1 g to 100 g using larger planetary ball mills, while sometimes successful,^25*b*^ often presented challenges. For example, it could cause the starting materials to be compacted into extremely hard, “rock-like” phases. As a consequence, the specific mechanical energy (SME), that is, the kinetic energy imparted per unit mass of reactants, no longer induced mixing, and the reaction did not occur. Moreover, as the scale of the equipment increases, the SME generally decreases, further reducing the likelihood of successful reaction progression.

Seeking to overcome the scale-up challenge, we turned to Professor Tony McNally, then based in the School of Mechanical and Aerospace Engineering at Queen’s University Belfast. He suggested Twin Screw Extrusion (TSE) as a solution. TSE is a high-throughput, low-volume processing technique in which material is forced through a confined space with simultaneous mixing, enabling not only blending but also potentially chemical reactions between solids—effectively and continuously at large scales.

By that time, TSE was already widely used in industry for blending materials, such as polymer composites or active pharmaceutical ingredients (APIs).^26^ Reactive extrusion was also established, mainly in the context of polymer chemistry (*e.g.* polymerization, depolymerization, grafting).^26^ Additionally, Paradkar at the University of Bradford had demonstrated hot melt synthesis of ibuprofen-nicotinamide co-crystals,^27*a*^ and Alvarez-Nunez at Amgen demonstrated melt and non-melt co-crystal synthesis.^27*b*^ Nevertheless, using twin screw extruders as reactors for covalent synthesis of small molecules, as might apply broadly in chemical manufacturing, remained largely unexplored.

**Fig. 1:**
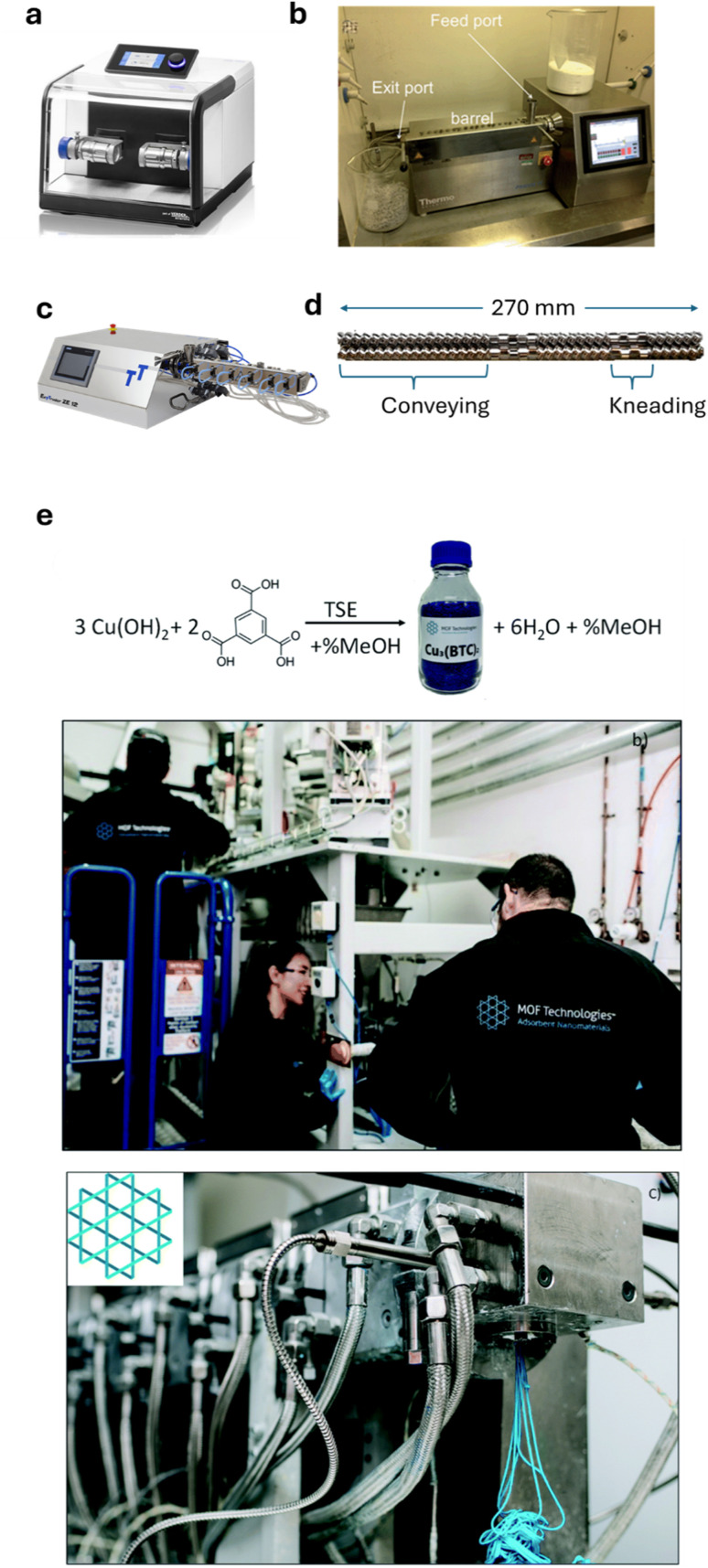
Overview of the mechanochemical technologies that have been employed in the synthesis of HKUST-1, from sub-gram batch scale to continuous kg h^−1^ scale. (a) Small shaker-style ball mill scale ball mill (Retsch MM400, reproduced with permission from ref. 11*c*), (b) Thermo Fisher Process 11 Parallel Co-Rotating twin screw extruder used in ref. 1 (reproduced with permission from ref. 1), (c) three-Tec ZE 12 twin screw extruder (Retsch MM400, reproduced with permission from ref. 11*c*), (d) typical screw configuration with a combination of conveying and kneading zones (reproduced with permission from ref. 1), (e) ton scale commercial production of MOFs directly in pellet form by Nuada (previously known as MOF Technologies) (reproduced with permission from ref. 29*a*).

Fig. 1 illustrates various equipment for batch and continuous mechanochemical synthesis. In TSE, material is conveyed forward by two intermeshing, co-rotating screws (or optionally counter-rotating) housed within a tightly fitting barrel. The screws are typically modular and the profile can be configured to include both conveying and kneading zones. The conveying zone, which primarily moves material along the barrel, also generates compressive forces as the extrudate is pushed against the barrel walls. The kneading zone, in which screw elements may be pitched at various angles, also contributes to compression and applies shear. This shear force has been shown to exert the greatest influence on mechanochemical reactions during reactive extrusion. Furthermore, in contrast to ball milling, TSE offers the advantage of precise temperature control, allowing materials to be heated to several hundred degrees Celsius or cooled if required. A die, commonly used in polymer processing and shaping applications (which can be an attractive aspect in synthesis by extrusion; see below), can be attached at the exit to increase back pressure and extend the residence time of the extrudate within the barrel, as well as shape the extrudate as it emerges.

Such extruders are not traditionally used with powders. However, several process parameters can be adjusted to provide effective process control, including screw rotation speed, feed rate (and therefore the fill level), screw configuration, and the temperature profile along the barrel. Each of these influences the residence time of material within the barrel. There is also potential to employ reactive or inert atmospheres such as CO_2_, H_2_, or N_2_, analogous to the use of supercritical CO_2_ in pizza dough processing by TSE. Although TSE is intrinsically continuous and often rapid because it employs neat reactants and elevated temperatures, inherently slow reactions can be accommodated by operating the process in a semi-continuous mode, as shown in the peptide formation study by Lamaty *et al.* (see below). This is not an exhaustive list of modifiable process parameters and, as research into continuous mechanochemistry continues to develop, further refinements are expected. For example, aspects discussed in the sections below include coating the screws with catalytic metals, or using alternative barrel materials to enable *in situ* X-ray diffraction techniques. More recently, continuous photo-extrusion has been demonstrated using a light-permeable resin to form the barrel.^27*d*^

Synthesis by extrusion can be seen as a kind of “flow chemistry without solvents”.^27*e*^ Solvent-based flow chemistry has become intensely researched in recent years for its advantages such as increased safety, control and sustainability, and is becoming more widely adopted in pharmaceutical and fine chemical synthesis.^27*e*^ Flow as a general technique has a longer history and is more developed than TSE. A huge opportunity therefore exists to apply concepts and practices from flow chemistry to synthesis by extrusion to accelerate its development and implementation.

For our first experiments, fortunately, QUB was equipped with a range of twin- and single-screw extruders in its Polymer Processing Research Centre. For lab-based synthetic chemists, it was unusual to bring kilograms of starting materials into a cavernous processing hall and feed them into the hoppers of these large machines, very different from the familiar round-bottomed flasks, autoclaves, or ball mills of the synthetic laboratory. Yet, after a few exploratory attempts to synthesise MOFs *via* TSE, the results were very promising. Remarkably, we could produce MOFs, and other products, quickly and in multi-kg quantities, all through this unconventional, industrial-style process. The scale and speed felt like opening a door to a new way of doing chemistry.

**Fig. 2:**
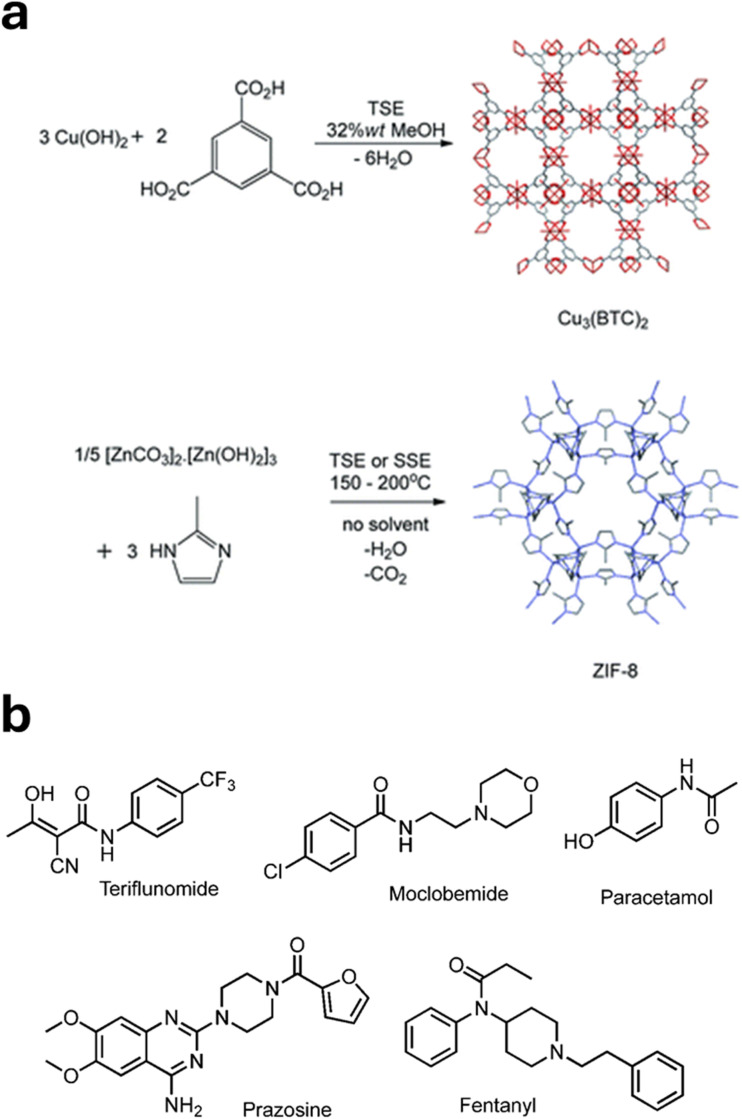
(a) Example reactions and structures for Cu_3_(BTC)_2_ and ZIF-8. (b) Example APIs made by TSE (reproduced with permission from ref. 1).

We developed synthesis protocols for three archetypal microporous MOFs—Cu_3_(BTC)_2_ (HKUST-1), ZIF-8 (MAF-4, Zn(methylimidazolate)_2_), and Alfum (Al(OH)(fumarate))—all of which were of potential commercial interest at the time. Fig. 2a shows example reactions and structures for Cu_3_(BTC)_2_ and ZIF-8. These methods enabled quantitative yields at rates of approximately 1.0 kg h^−1^. Remarkably, the corresponding Space Time Yields (STYs, the amount of product per unit reactor volume per day) ranged from 27 000 to 144 000 kg m^−3^ day^−1^, representing 2–3 orders of magnitude greater than conventional batch syntheses in some cases. STY is an indicator of process intensity, with higher values reflecting greater overall efficiency. The substantial improvement in STYs arose from the combination of low reactor volume, high product throughput, and the use of neat reactants, which allowed fast conversion rates in the absence of solvents. Further scalability was demonstrated through the solid-melt synthesis of ZIF-8 using a single-screw extruder (SSE) at a throughput of approximately 4.0 kg h^−1^. Overall, this work clearly demonstrated the potential of extrusion-based mechanochemical synthesis for continuous, commercial-scale production of MOFs involving covalent bonds. Consequently, we acquired a compact lab-scale twin screw extruder (Three-Tec ZE12) that could fit inside a fume cupboard, enabling us to carry out more detailed studies and further expand the scope of TSE in mechanochemical synthesis.

## Further scale-up and commercialisation of synthesis by TSE

MOF synthesis by TSE was further developed by the QUB spin-out company MOF Technologies Ltd, since rebranded as Nuada,^28^ which provided MOFs in ton quantities by TSE. Nuada uses these MOFs in pilot-scale industrial carbon capture units, each capable of capturing approximately 1 ton of CO_2_ per day. The physisorption mechanism of CO_2_ capture in these materials delivers energy savings compared with the chemisorption processes used in conventional aqueous amine systems. Beyond its intrinsic low-solvent efficiency, an additional advantage of the TSE MOF production method is the ability to combine synthesis and processing into a single operation. With appropriate control of processing conditions, MOFs can be produced directly not as powders but in pellet form (and without the need for post-synthetic washing). This is an important attribute of TSE, as powder forms are often problematic in practical applications.^29*a*^ Related to this, Wang *et al.* have shown that MOFs can be synthesized by TSE in the presence of a polymer binder to give MOF-polymer composites as pellets directly.^29*b*^ Although it is impossible to know everything that occurs in industry, the Nuada TSE MOF manufacturing process is, to our knowledge the first, and currently most advanced commercial application of mechanochemical synthesis. Its deployment at industrial scale provides compelling evidence that mechanochemistry can indeed move beyond the laboratory and deliver viable manufacturing solutions, making it an important milestone in the field.

## Growth of synthesis by extrusion, 2015–present

Since those early demonstrations, the field has developed rapidly, with numerous groups expanding, deepening understanding, and refining the use of TSE across diverse areas of chemical synthesis. Some key developments are summarised below.

### Extension to other porous materials

Karadeniz *et al.* extended this work to Zr-based MOFs, in particular UiO-66-NH_2_.^30^ This is noteworthy because such MOFs, constructed from less labile, more oxophilic metal ions, are highly valued for their stability and yet are often more challenging to prepare in a highly crystalline form. Their strategy made use of Zr_6_ oxo-acetate clusters as reactants, effectively serving as pre-formed network nodes. Importantly, the resulting materials exhibited high surface areas without requiring a washing step, making the overall process highly efficient. Metal-nitrate-4,4′-bipyridine networks have also been successfully prepared by TSE, as reported by Zaworotko and Walker *et al.*, who concluded that mechanosynthesis of these materials provides similar outcomes to solvent-based crystallisation. The authors also explored water-slurry synthesis of these water-stable, water-insoluble coordination polymers, anticipating that it might offer a high process-intensity route. In practice, however, that approach proved ineffective, particularly when compared with the mechanochemical methods reported.^31^

TSE has also been shown to be applicable to fully organic porous materials. Notably, Banerjee *et al.* extended the technique to the synthesis of covalent organic frameworks (COFs) based on imine linkages.^32^ Their work employed a ‘microcompounder’, equipped with conical screws rather than the conventional cylindrical configuration used in standard extrusion equipment. This type of screw extrusion apparatus has since been adopted more widely, including in organic synthesis and active pharmaceutical ingredient (API) manufacture (see below). In addition, 10-component porous coordination cages linked by imine groups have been synthesised by TSE; interestingly, this process also led to partial formation of a kinetically trapped 8-component “partial cage” structure.^33^

### Organic synthesis

Demonstrating the applicability of TSE to the covalent synthesis of purely organic small molecules was another important milestone, opening up opportunities for the production of pharmaceuticals, agrochemicals, dyes, and beyond.

Initially, we showed that a range of condensation reactions could be carried out efficiently using TSE, often at temperatures below the melting points of the reagents.^34^ This suggested that, at the bulk level, the processes operated under solid-state conditions rather than as conventional hot-melt extrusion (HME). However, even within these seemingly simple reactions (Knoevenagel condensations, Michael additions, and aldol reactions) we quickly began to appreciate the importance of process chemistry and the physical properties of the reagents during extrusion.

In studying the Knoevenagel condensation, a benchmark reaction in mechanochemistry, discussions with Prof. Gavin Walker (University of Limerick) led us to recognise the key role of wettability. We initially explored the correlation of reactivity with Hammett parameters amongst various aromatic aldehydes, expecting electronic effects to determine reactivity. Instead, all reactions were found to require a similar temperature of 160 °C to reach full conversion. Contact-angle measurements of the aldehydes on barbituric acid revealed that optimal wettability was only achieved at this temperature, suggesting that physical properties related to reagent mixing were critical. Although a melt phase was clearly important for reactivity, the material still appeared macroscopically solid throughout the process. In most cases, these condensation reactions proceeded rapidly, achieving excellent conversions in as little as 2 minutes, particularly once the key physical aspects of the systems were understood. In such reactions, the combination of neat reagents (high concentrations) and elevated temperatures was crucial in enabling these remarkably short residence times. However, not all systems behaved in this manner. For example, the Michael addition between veratraldehyde and dimedone was slower, and wettability did not appear to be a dominant controlling factor. To address this, we modified the screw design by incorporating “reverse” screw elements, which retard the flow of material. Strategically positioned after the kneading zone, these elements extended the residence time under high shear while maintaining constant screw rotation speed, ultimately enabling efficient conversion in these more demanding systems.

TSE has also enabled more complex condensation reactions, such as the synthesis of commodity perylene dyes from anhydrides and primary amines.^35^ Unlike conventional batch processes, TSE avoids harsh conditions, long reaction times, and problematic solvents like DMF, offering a greener and more efficient manufacturing process (as discussed in the Life Cycle Analysis below). In several cases, notably the synthesis of pigment Black 31, thermal activation of the reagents is essential for high conversion, while TSE provides superior mixing.

Amide bond formation is one of the commonest reactions in pharmaceutical synthesis but can be challenging to achieve. In a comprehensive study, Browne *et al.* successfully obtained 36 amide products by TSE from a range of solid–solid, solid–liquid, and liquid–liquid reactions starting from esters, using both inorganic and organic bases to catalyse the reactions.^36^ Notably, the study included a 7-hour run with automated feeders, producing approximately 0.5 kg of amide product, demonstrating the robustness of TSE for chemical synthesis. The work also highlighted the value of a solid-state grinding auxiliary in facilitating liquid–liquid reactions, likely acting as a support that enables mechanical energy, through shear and compression, to be applied more effectively.

Métro, Lamaty, and co-workers used an Xplore Pharma Melt Extruder (PME) in the synthesis of di- and tri-peptides, including the artificial sweetener aspartame, *via* amide bond formation from carboxylic acids and amines, using coupling agents such as diisopropylcarbodiimide and *N*,*N*′-dicyclohexylcarbodiimide.^37*a*^ Remarkably, these reactions can be carried out under solvent-free, CMR-free (Carcinogenic, Mutagenic, or Reprotoxic) extrusion conditions, yielding sequence-specific peptides.^37*b*^ Hernández *et al.* demonstrated enzyme-catalysed synthesis of amino acids into oligopeptides using papain. Remarkably, the enzyme not only withstood the mechanical forces of TSE but could also be recovered and reused, highlighting the robustness of enzymes under extrusion conditions. Using moderate TSE settings (30 rpm, 50 °C), oligopeptides with a degree of polymerization of 5.4 were obtained. Moreover, the open-reactor nature of TSE helped manage the humidity critical for this hydrophobic enzyme, allowing water vapour to escape while maintaining temperature control and optimal reaction conditions.^37*c*^

Significant advances in synthesis by TSE have come from the work of Browne with the difluorination of dibenzoylmethane using Selectfluor, scaled from ball milling to TSE, which increased the STY 100-fold, from 29 to 3395 kg m^−3^ day^−1^.^38^ The study highlighted two key insights: first, selectivity could be finely controlled (83% difluorinated product at 70 °C using a reverse screw configuration with 10 min residence time at 280 rpm), and second, the required base loading dropped from 10 mol eq. to 3 mol eq. This work illustrated the critical interplay between mechanical energy, screw speed, and residence time, with TSE offering a platform to optimise reaction efficiency. Further refinements included LAG to favour monosubstitution and the use of NaCl as a glidant to improve extrudate flow, particularly in systems with challenging rheology or limited reagents. Collectively, these advances underscore TSE’s versatility for controlling reactivity, selectivity, and scale, cementing its role in modern mechanochemical synthesis.

Leitch and Browne *et al.* undertook pioneering efforts to translate ball-milling protocols to TSE, demonstrating for the first time that mechanochemical reaction design principles could be successfully implemented in a continuous-flow format. This was exemplified by Ni-catalysed C–C coupling reactions conducted in a TSE using NiCl_2_(PPh_3_)_2_, employing a stepwise barrel-heating profile designed to emulate optimised ball-milling conditions.^39*a*^ Building on this foundation, Hastings, Co, and Speight further established the viability of metal-catalysed synthesis under TSE conditions by demonstrating a Pd-catalysed Sonogashira coupling using Pd(PPh_3_)_4_.^39*b*^ Notably, and unusually for TSE studies, this work demonstrated the transferability of the chemistry across two distinct extruder platforms (Leistritz Nano 16 and Thermo Fisher Process 11), highlighting the robustness and broader applicability of TSE-enabled synthesis. With regard to metal-catalysed reactions, Rodriguez-Padrón and Selva *et al.* have shown that Pd deposited on *N*-doped carbon was an effective “heterogeneous” catalyst for the Suzuki–Miyaura cross-coupling reaction of iodobenzene and phenylboronic acid in an extruder. Notably, the catalyst was itself made by an extrusion process starting from Pd(OAc)_2_ and chitin.^39*c*^ Kulkarni *et al.* have developed a vertical, single-screw reactor (PTFE) with a glass jacket with the ability to transport cooling or heating fluids around the outside of the reaction chamber. As a result, the authors have been able to demonstrate reactions involving highly corrosive reagents, such as nitric acid for the nitration of aromatics.^40^

### TSE in medicinal chemistry

Demonstrating the industrial viability of mechanochemistry requires showing that it can effectively and efficiently deliver commercially relevant products. Building on previous work on the commercial synthesis of MOFs by TSE, collaborative work with Colacino *et al.* demonstrated the first API synthesis by TSE – the antibiotic nitrofurantoin at the 25 g scale in high purity – while also indicating (see below) the potential for reduced carbon emissions and cost.^41^ Extending this approach, Lavayssiere and Lamaty used their Xplore Pharma Melt Extruder in semi-continuous mode to synthesise amide and peptide APIs, successfully producing Teriflunomide, used for multiple sclerosis, and Moclobemide, used for depression.^42^

More recently, using their bespoke glass-jacketed screw reactor, Kulkarni *et al.* demonstrated the final step of a four-step continuous synthesis of the leishmaniasis drug miltefosine.^43^Fig. 2b shows example APIs made by TSE.

Collectively, these examples underscore the applicability of TSE in pharmaceutical synthesis. TSE is already used in GMP-compliant API formulation, alleviating concerns about contamination of products from contact with steel screws and barrels. Furthermore, pharma-grade 316L stainless steel, as used by manufacturers such as Three-Tec, has excellent chemical resistance, further supporting the suitability of TSE in this context.

## Advancing TSE to address the broader demands of chemical manufacturing

While substantial progress has been made in performing chemical reactions by TSE, and it has been implemented commercially for MOF production, its transition into a broad-based manufacturing platform will depend on its ability to address wider processing challenges and enable increasingly sophisticated production strategies. These include the synthesis of liquid products, the immobilisation of catalysts on extruder reactor surfaces, and other emerging approaches discussed below.

### Deep eutectic solvents, DESs

Deep eutectic solvents (DESs) are a versatile class of multicomponent solvents with a wide range of potential applications, including electroplating.^44^ Their large-scale production by batch methods can be challenging due to the high viscosity of the liquid phase and the slow dissolution of solid components, which often requires prolonged heating. With phase changes occurring in batch processes, DES components can become immobile, necessitating slow addition over 12 hours with continuous stirring and heating, which severely limits throughput. In carbohydrate-based DESs, this can further lead to partial decomposition (caramelisation). TSE has been shown to overcome these limitations: three archetypal DESs were formed rapidly with minimal decomposition, requiring only brief heating as the mixture passed through the barrel. Space-time yields were up to four orders of magnitude greater than for conventional batch methods, highlighting that TSE is not only applicable to solid products but also to liquid chemical syntheses.^45^

### Combining synthesis and processing into a single operation

Chemical manufacturing typically involves multi-step processes, and the applicability of TSE therefore depends on its ability to accommodate such complexity. This has been demonstrated previously through the synthesis and direct formulation of MOFs into pellets,^29^ and more recently extended to multistep organic transformations, including Michael–Aldol reactions and the combined synthesis and amorphisation of paracetamol.^46^ Specifically, paracetamol was synthesised from acetic anhydride and 4-aminophenol to afford the API in its crystalline form; however, by introducing an equimolar amount of citric acid into the reactants, the product could be formed and simultaneously rendered amorphous. In this context, citric acid acts as a co-former that inhibits crystallisation, an approach of particular relevance to pharmaceutical applications where amorphous APIs can exhibit enhanced kinetic solubility.^47^

### Mechanocatalysis with coated screws/barrel

Earlier pioneering work by Mack showed that in ball milling the milling medium itself can act as, or be a source of catalyst, for example in the use of copper balls/jars in combination with Pd(PPh_3_)_4_ for the Sonogashira reaction.^48^ This phenomenon has become known as ‘direct mechanocatalysis’. This idea has been successfully extended to TSE by Borchardt *et al.* who electrocoated portions of the steel screws with layers of Cu, Ni, Au and finally Pd and showed that the extruder could then be used to catalyse Suzuki–Miyaura reactions, resulting in a STY of 82 kg per day per m.^49^ Given the importance of transition metal catalysts in organic synthesis, there is clearly much potential for exploring a wide range of other metals and reactions and thereby increasing still further the efficiency and scope of synthesis by TSE.

### Delamination – synthesis of 2-D materials

The fact that TSE imparts high shear led to the idea that it might be applied not only to constructive synthesis but also to ‘destructive’ synthesis, such as delamination of layered materials. As a proof of concept targeting the archetypal two-dimensional material graphene, TSE was shown to delaminate graphite to produce multilayer graphene (*ca.* 6 layers).^50^ The optimised process built upon previous work by ball milling, and made use of aromatic additives such as pyrene (which were liquid at the temperatures used) to facilitate the extrusion process with NaCl acting as a solid, physical grinding aid. The process can therefore be described as solid-and-liquid-assisted extrusion (SLAE). The process had a number of benefits including continuous production and relatively high yield compared to some other methods for delamination of graphite. It is possible therefore that TSE could be a general technique for delamination of other 2-D phases such as hexagonal BN, MoS_2_*etc.*

### *In situ* monitoring

As mentioned above, *in situ* spectroscopic monitoring has been important and enlightening for understanding mechanochemical synthesis by ball milling. With regard to extrusion, *in situ* monitoring is potentially a still more challenging operation. Emmerling and co-workers have done pioneering and elegant work to successfully demonstrate *in situ* monitoring by both Raman spectroscopy and energy-dispersive X-ray diffraction (EDXRD).^51,52^*In situ* Raman monitoring enabled the optimisation of the TSE-synthesis of ZIF-8 with regard to added liquid (ethanol) and reactant stoichiometry, leading to a process which gave high quality product and with improved efficiency and sustainability. *In situ* EDXRD was demonstrated on four different reaction systems (a salt metathesis, MOF synthesis and two cocrystal syntheses) which enabled the formation of kinetic intermediates and side phases to be identified. *In situ* monitoring is likely to gain importance towards developing kinetic models for extrusion-based reactions.

## How sustainable and cost-effective is it?

The attractiveness of mechanochemical synthesis as a route to more sustainable manufacturing has long been long recognised.^15^

**Fig. 3:**
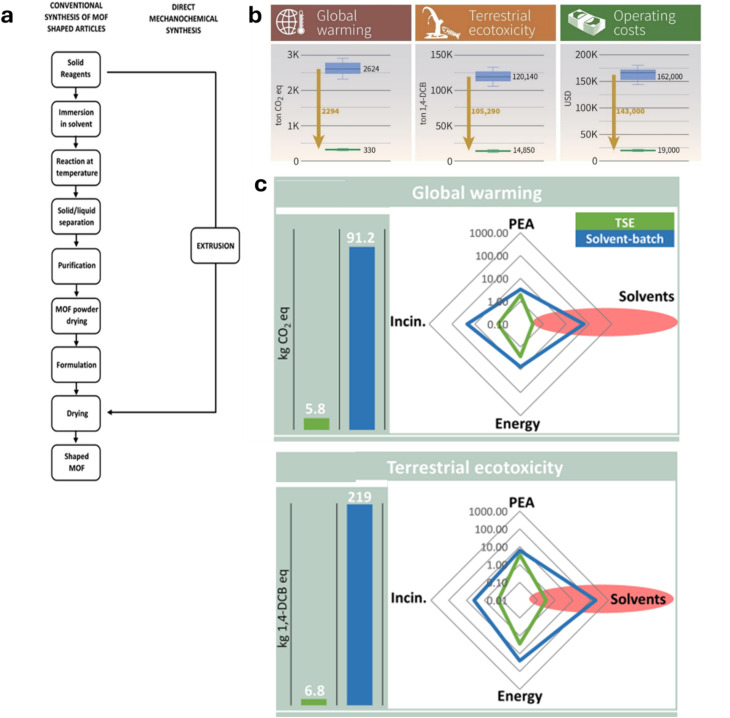
Illustrates how TSE can make chemical synthesis significantly more efficient, sustainable from an environmental perspective, and less costly: (a) the dramatic shortening and simplification of MOF manufacturing (synthesis and shaping) by extrusion compared to conventional solution methods (reproduced with permission from ref. 29*a*). (b) Life Cycle Assessment (LCA) for the synthesis of the antibiotic nitrofurantoin by extrusion compared to conventional solvent batch synthesis showing order of magnitude or greater reductions in global warming, ecotoxicity and cost indicators (reproduced with permission from ref. 54). (c) Results of LCA for manufacturing a perylene dye (Black-31) showing the order-of-magnitude (or greater) reduction in global warming and terrestrial ecotoxicity indicators by switching from conventional solvent-batch (blue) to continuous solvent-free synthesis by TSE (green) (reproduced with permission from ref. 54).

The flow diagram in Fig. 3a showing the process steps involved in MOF production taken from Casaban *et al.*^29*a*^ provides a clear and striking illustration of how much more efficient TSE can be compared to solvent-batch methods. However, quantification of mechanochemistry’s sustainability benefits is still surprisingly sparse. Two notable life cycle assessments (LCAs) have been done comparing TSE synthesis to solvent-batch methods for the production of nitrofurantoin,^53^ and a perylene dye, Black-31,^54^ both by Spatari *et al.* Remarkably, compared to traditional solvent-based batch procedures, it was found that using TSE reduced environmental impact by around an order of magnitude, according to various sustainability indicators such as carbon footprint, eco-toxicity *etc.* Furthermore, even the cost of the API synthesis by TSE was predicted to be reduced by a similar degree compared to the reported solvent batch method. Notably, for the dye synthesis, the requirement for use of a solvent (MeOH) to purify the product was included in the assessment and even then order-of-magnitude improvements in sustainability were still found. This speaks volumes to a sometimes-perceived limitation of mechanochemistry, that it only reduces or eliminates solvent usage for the synthesis step and that solvents are still needed for purification. This aspect has been discussed and addressed in a general sense in the 2012 *Chemical Society Reviews* article,^15^ but the dye LCA work shows that even if solvents are needed for purification, the sustainability benefits of TSE can still be remarkable.

## Rheology and rheokinetics in mechanochemical synthesis

Elucidating the unusual rheological changes that occur during mechanochemical synthesis is critical to understanding and predicting reaction behaviour, ultimately de-risking scale-up and facilitating wider industrial implementation.^22,55^ As discussed above, the kinetics of solvent-free ball-milling reactions are often intimately linked to changes in the rheology of the reaction mixture. A well-known example is the transition from a free-flowing powder to a transient rubbery “cohesive state”, during which the reaction accelerates, followed by reversion to a powder product, giving rise to sigmoidal kinetics.^22^ During synthesis by extrusion, analogous behaviour is observed. As reactions initiate, the screw torque, the rotational force required to maintain a constant screw speed, frequently increases, reflecting reaction-induced changes in viscosity. This phenomenon was quantified using a twin-rotor batch mixer designed to mimic a static zone within a twin-screw extruder.^56^ Multi-gram reactions between solid metal acetate salts and the chelating ligand 8-hydroxyquinoline were studied, yielding metal complexes of interest for OLED applications. An initial induction period characterised by low torque was observed, during which little or no reaction occurred, followed by pronounced torque increases associated with rising viscosity of the reaction mixture. Notably, these torque responses coincided with the onset of reaction.^56^

Rheokinetic analysis such as that described above is well established as a cornerstone of polymer chemistry and processing.^57^ The studies noted here demonstrate that the same is likely to apply to mechanochemical synthesis. As the field matures, rheokinetic analysis will probably become critical for achieving predictive understanding, robust process control, and reliable scale-up of mechanochemical and extrusion-based reactions.

## Translating smoothly from batch (ball milling) to continuous (TSE) mechanochemistry

The ability to translate smoothly from batch mechanochemistry (*e.g.* ball milling) to extrusion is important. For example, reaction conditions can be screened using reactants at small scales (less than one gram) by ball milling rather than having to use large amounts of material for trial extrusion runs (often tens of grams). How various parameters from each method might interrelate has been explored by us,^34^ Browne *et al.*^38^ and Hastings, Co and Speight *et al.*^39*b*^ Also, use of the twin-rotor batch mixer^56^ mentioned above showed how such equipment with its torque data readout and the ability to vary fill level, temperature and rotor speed could be used successfully to guide the choice of conditions for successful synthesis by extrusion.

Andersen *et al.* showed that where temperature-controlled heating could be incorporated into a ball mill, this provided a helpful platform for predicting reactivity under TSE. Conversions in each type of apparatus corresponded well to each other at given temperatures, although conversions for TSE tended to be a little higher. This was demonstrated for S_N_Ar and Knoevenagel reactions. S_N_Ar reactions are important in pharma and agrochemical synthesis but in conventional solvent-bath mode use large amounts of polar aprotic solvents which is undesirable.^58^

In addition, although many ball mills lack temperature control, it has also been shown that a “beat-and-heat” approach in which a period of ball milling is followed by heating the milling jar and contents in an oven, can also give effective guidance on moving to TSE.^35^

## Conclusions and outlook

Since the publication of the 2015 paper,^1^ synthesis by TSE has progressed substantially, even gaining recognition from IUPAC as one of ten technologies with the potential to change the world.^59^ It has emerged as the leading approach for the scale-up and industrial implementation of mechanochemical synthesis, exemplified by the commercialisation of MOF production, while also demonstrating at smaller scales broadening applicability across chemical synthesis. At the same time, understanding of reaction behaviour under TSE continues to advance, supported by *in situ* monitoring and growing mechanistic insight. Coupled with its advantages in cost, sustainability, and compatibility with GMP manufacturing, these developments position TSE well for wider adoption in industrial chemical synthesis, including the pharmaceutical and agrochemical sectors in due course if embraced broadly, TSE has the potential to impact positively on the sustainability and general development of chemical manufacturing.

Despite this progress, important opportunities and challenges remain. A key priority is establishing reliable routes to translate mechanochemical processes from laboratory and pilot scales to industrial operation, particularly for solvent-free, non-melt systems that consist of several phases (solid, liquid, gas) and may behave quite differently from conventional polymer melts. Additional opportunities include tethering catalysts to extruder surfaces to create continuous solid-state catalytic reactors, adapting TSE for the safe handling of hazardous materials, and reducing solvent dependence through the development of solid-state purification strategies. Challenges also remain in process screening and optimisation, which currently require relatively large quantities of material. Addressing these areas will be critical to fully realising the industrial potential of TSE.

## Author contributions

DEC and SLJ drafted and revised the manuscript jointly.

## Conflicts of interest

SLJ is a shareholder in Nuada Ltd.

## Data Availability

There is no additional data associated with this article.
